# Novel Passive Two-Stage Magnetic Targeting Devices for Distal Locking of Interlocking Nails

**DOI:** 10.1155/2017/3619403

**Published:** 2017-08-13

**Authors:** Tze-Hong Wong, Meng-Shiue Lee, Sung-Yueh Wu, Wensyang Hsu, Tien-Kan Chung, Chia-Pei Wu, Pei-Jung Hsu, Yuh-Shyong Yang

**Affiliations:** ^1^Department of Biological Science and Technology, National Chiao Tung University, Hsinchu 30010, Taiwan; ^2^Department of Orthopedics, National Taiwan University Hospital Hsinchu Branch, Hsinchu 30059, Taiwan; ^3^Department of Mechanical Engineering, National Chiao Tung University, Hsinchu 30010, Taiwan; ^4^Additive Manufacturing and Laser Application Center, Additive Manufacturing Innovation Department, Industrial Technology Research Institute, Tainan 73445, Taiwan

## Abstract

Interlocking nailing is a common surgical operation to stabilize fractures in long bones. One of the difficult parts of the surgery is how to locate the position and direction of a screw hole on the interlocking nail, which is invisible to the naked eye after insertion of the nail into the medullary canal. Here, we propose a novel two-stage targeting process using two passive magnetic devices to locate the position and direction of the screw hole without radiation for the locking screw procedure. This involves a ring-shape positioning magnet inside the nail to generate a magnetic field for targeting. From the accuracy test results of these two-stage targeting devices, the search region can be identified in less than 20 seconds by the 1st-stage targeting device, while the total targeting time to locate the drilling position and direction takes less than 4 minutes, with 100% successful rate in 50 attempts. The drilling test further combines the two-stage targeting process and drilling process on the swine tibia, and it is shown that a 100% successful rate is achieved in all 10 attempts, where the total time needed is less than 5 minutes.

## 1. Introduction

**Figure 1:**
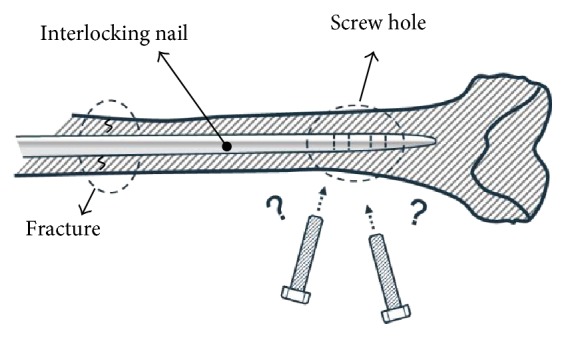
Distal locking of interlocking nail.

Interlocking nailing, also known as intramedullary nailing, is a common surgical operation to stabilize fractures in long bones [1–5] and is one of the best methods for treating fractures of the lower extremities [6, 7]. The procedure involves the insertion of a hollow nail in the bone medullary canal, which is secured by screws at the proximal and distal ends to prevent the rotation or displacement of the bone after adequate reduction. One of the most difficult parts of the surgery is to find the accurate drilling position and screwing direction for the interlocking screw, which is invisible to the naked eye after the insertion of the nail into the long bone, as shown in Figure 1.

The target-aiming devices based on the parallel mechanism [4, 8–10] have been clinically used, but they often fail to provide an accurate drilling location due to nail distortion during insertion. X-ray imaging is a direct method to locate screw hole orientation through radiological imaging, so-called the free-hand method [11, 12]. Utilizing X-ray-imaging approach, however, exposes surgeons, medical teams, and patients to radioactivity hazard for a considerable dose. To avoid using X-rays, which are harmful to humans, transilluminating [13], sound-guided [10], ultrasonic [14], and magnetic [15, 16] methods were proposed to find the drilling position. However, these methods were unable to locate the exact drilling direction. A magnetic assistant system [17–19] with magnetic sensors was proposed for both positioning and directional guidance, but it lacked accuracy and required complicated signal processing.

In this study, a novel two-stage magnetic targeting process with two passive targeting devices is proposed to provide a rapid and accurate method for the distal locking of interlocking nailing without radiation. The 1st-stage targeting device is used to focalize the screw hole area rapidly for the next targeting process. The 2nd-stage targeting device is used to identify accurately not only the position but also the direction of the screw hole of the interlocking nail. Furthermore, a light-based indicator is integrated with the 2nd-stage targeting device to indicate the alignment state during the targeting process in a more intuitive way.

## 2. Concept Design

**Figure 2:**
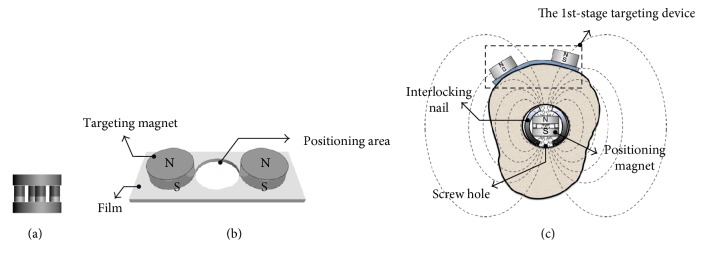
Schematic diagrams of (a) the positioning magnet, (b) the 1st-stage targeting device, and (c) the 1st-stage targeting device attracted by the positioning magnet.

To ensure a radiation-free method in the distal locking process for the interlocking nailing operation, passive magnetic sensing is chosen for targeting. It is known that the magnetic field can pass through human tissue without damaging the tissue or undergoing significant distortion, so it is suitable for biomedical applications [17]. To apply magnetic field targeting, the magnetic field from a permanent magnet inside the nail is used as a positioning magnet, where the position and magnetic field direction of the magnet are detected remotely through the magnetic guiding device outside the bone. The positioning magnet is made of neodymium iron boron (NdFeB), which is biocompatible and can provide a strong magnetic field for targeting. The proposed shape of the magnet is cylindrical with a hollow hole at the center, as shown in Figure 2(a); the magnetic field gradient is concentric and uniform around the magnet and the flux lines converge on the center of the magnet. Here, the positioning magnet is placed on the screw hole and the magnetic field direction is aligned with the drilling direction, after which the positioning magnet can determine the drilling position and direction.

### 2.1. The 1st-Stage Targeting

The 1st-stage targeting device consists of two permanent magnets and a flexible thin film. The two permanent magnets, called targeting magnets, are fixed on the flexible thin film in the same magnetic direction, as shown in Figure 2(b). When a positioning magnet is inside the cylindrical limbs, the targeting magnets are rapidly attracted to the skin surface by the positioning magnet, but two targeting magnets are also repelled each other in static equilibrium due to the cylindrical geometric condition, as shown in Figure 2(c). The screw hole area can then be focalized rapidly to a definitive region between the two targeting magnets. An opening between two targeting magnets on the film is designed so that the area can be easily marked for the next targeting stage.

### 2.2. The 2nd-Stage Targeting

**Figure 3:**
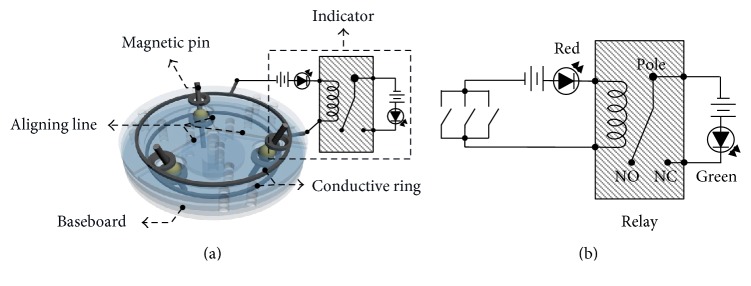
Schematic diagram of (a) the 2nd-stage targeting device and (b) equivalent circuit of the 2nd-stage targeting device.

The 2nd-stage targeting device consists of a transparent baseboard with three aligning lines, conductive rings, three magnetic pins with rotary joints, and an indicator, as shown in Figure 3(a). The three symmetrically placed magnetic pins can rotate freely and point to the positioning magnet inside the nail. The three magnetic pins act as three mechanical switches in operation, which are linked to the indicator by conductive rings and used to indicate alignment or misalignment by a light signal. Each magnetic pin can rotate to the strongest gradient direction of the nearby magnetic field from the positioning magnet inside the nail. The magnetic pin has two equal parts with a rotary joint in the middle to eliminate the gravity effect in operation, which can provide a flexible and unrestricted operation method in surgery. The transparent baseboard with three aligning lines provides a clear field of vision to observe the motion of the magnetic pins, which is used to guide the device to rapidly and accurately identify the alignment status. The indicator consists of two light-emitting diodes (LEDs) and a relay with DC voltage through a simple circuit, as shown in Figure 3(b), to indicate the alignment (green light) or misalignment (red light) state directly. The LED signal can help the user to determine alignment state in a more intuitive way.

**Figure 4:**
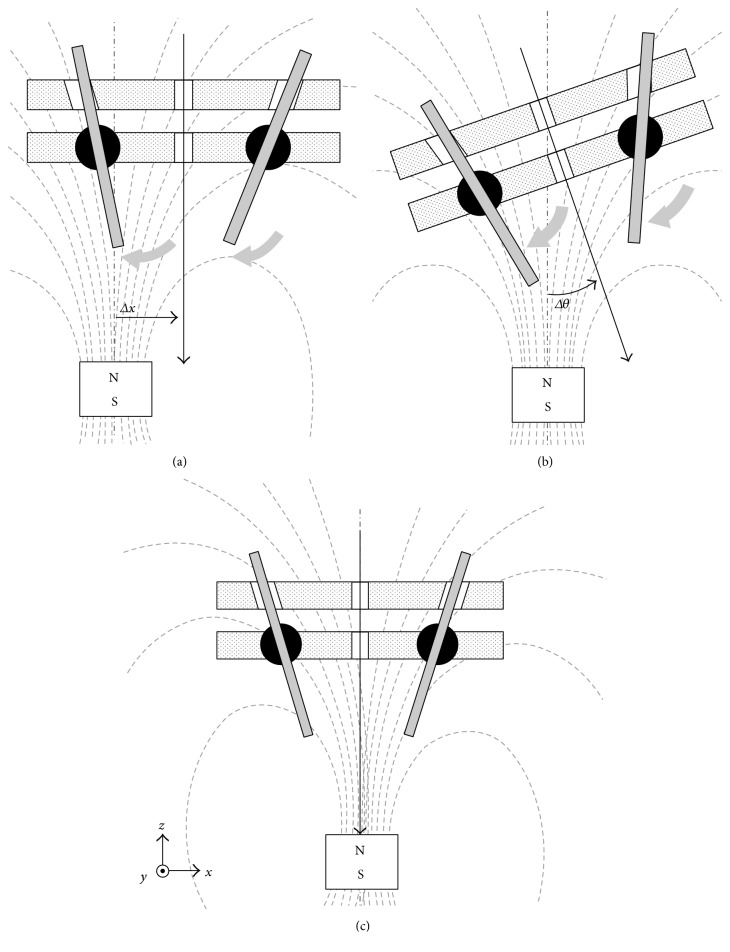
Illustrations of the 2nd-stage targeting device: (a) misalignment due to incorrect position for pins to contact with the conductive ring, (b) misalignment due to incorrect direction for pins to contact with the conductive ring, and (c) correct alignment.

In the proposed design, when the 2nd-stage targeting device misaligns with the positioning magnet either in position or direction, one or more pins will contact with the conductive ring on the transparent baseboard to turn on the red LED, as shown in Figures 4(a) and 4(b), respectively. By moving the guiding device around the positioning area identified by the 1st-stage targeting device, once three magnetic pins reach to symmetrical positions without contacting the conductive ring on the upper transparent baseboard, as shown in Figure 4(c), the green LED instead of red will be switched on. The 2nd-stage targeting device is considered to be aligned with the positioning magnet inside the nail. Then, the drilling process can be performed through the drilling hole at the center of the 2nd-stage targeting device.

**Figure 5:**
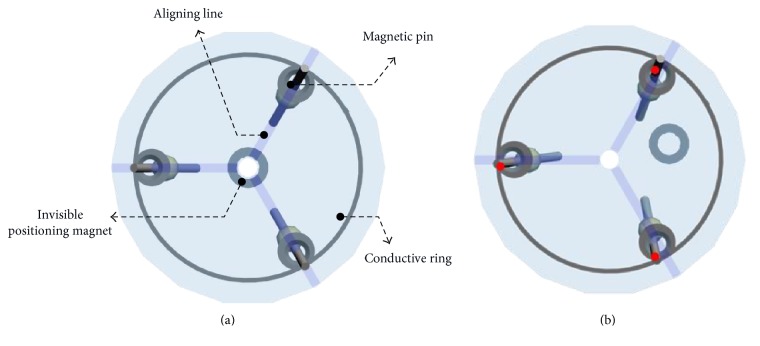
Alignment observation through deviation between pins and three aligning lines on the 2nd-stage targeting device: (a) correct alignment and (b) deviation between pins and aligning lines due to incorrect position or direction.

The transparent baseboard with alignment lines can provide a clear field of vision to observe the motion of the magnetic pins. When the guiding device aligns with the invisible positioning magnet in the nail, the projection of the three magnetic pins should overlap the aligning lines, as shown in Figure 5(a). When the guiding device misaligns with the magnet either in position or direction, one or more pins will deviate from the aligning line on the transparent baseboard, as shown in Figure 5(b). Then, the adjustment of the device should be continued.

## 3. Detail Design

Due to the space limit of the interlocking nail, the positioning magnet made of NdFeB (N52, Asia Magnets Co. Ltd.) with a 9 mm outer diameter, a 5 mm inner diameter, and 7 mm high is designed to place into a commercial interlocking nail with inner radius of 10 mm and outer radius of 12 mm. The following designs and tests on the 1st-stage and the 2nd-stage targeting devices are all based on this positioning magnet. However, the maximum operating temperature of the positioning magnet used in this work should be less than 140°C [20, 21]. Therefore, in the sterilization process of the positioning magnet, the operating temperature should be less than 140°C to main the strength of positioning magnet. For example, some clinical low-temperature sterilization processes, such as EO sterilization, activated glutaraldehyde sterilization, and plasma sterilization, are operating below 140°C.

### 3.1. The 1st-Stage Targeting Device

**Figure 6:**
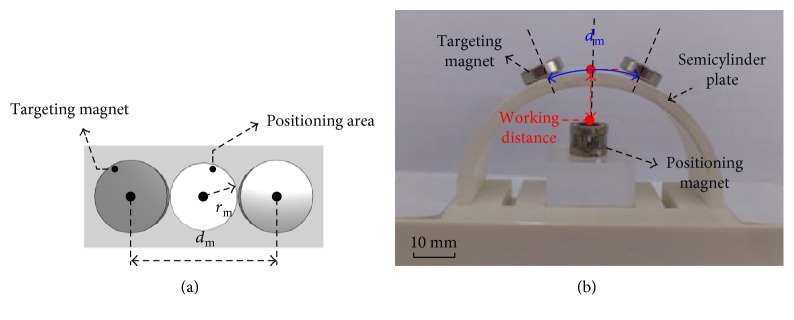
Critical dimensions of the 1st-stage targeting device: (a) the distance between two targeting magnets is *d*_m_ and the radius of the position area is *r*_m_ and (b) experimental setup for determining *d*_m_.

For the 1st-stage targeting device, two critical parameters need to be identified: the distance between two targeting magnets (*d*_m_) and the radius of the positioning area (*r*_m_), as shown in Figure 6(a). Here, these two parameters are decided experimentally. The experimental setup consists of the positioning magnet, two targeting magnets, and a semicylinder plate with a radius of 30 mm, as shown in Figure 6(b). The two targeting magnets, with radius of 4.5 mm and thickness of 2 mm, are also made of NdFeB. The semicylinder plate with radius of 30 mm is used to simulate the geometry of the limb. Since the minimum radius of the limb is 16 mm, the working distance between targeting magnet and the positioning magnet would be 16 mm at least. Then, the following steps are performed: (i) fixing the positioning magnet above the center of the semicylinder plate, (ii) placing two targeting magnets on the semicylinder plate, and (iii) measuring the distance between the two targeting magnets. For the working distance of 16 mm, the measured distance between two targeting magnets is in the range of 12 mm to 20 mm. Therefore, 20 mm is chosen to be the distance of two targeting magnets (*d*_m_) on the flexible film. Also, the shortest distance between the edges of two targeting magnets is about 11 mm due to 9 mm diameter of the targeting magnet. Therefore, 5 mm is selected to be the radius of the hollow circle, *r*_m_, for positioning area.

### 3.2. The 2nd-Stage Targeting Device

**Figure 7:**
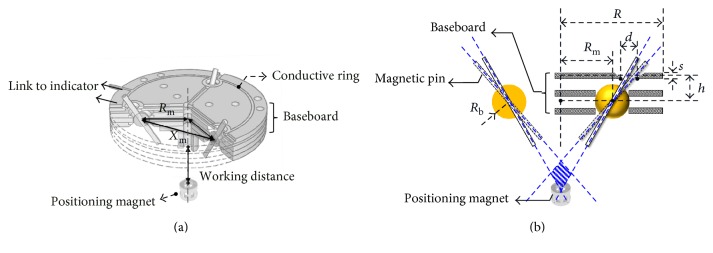
Illustrations of parameters in the 2nd-stage targeting device: (a) the distance from the board center to the rotary joint of magnetic pin (*R*_m_) and the distance between two magnetic pins (*X*_m_) and (b) other related parameters.

For the 2nd-stage targeting device, there will be three symmetrically placed magnetic pins on the transparent baseboard. Therefore, the critical parameter is the location of the pin on the baseboard, that is, the distance from the rotary joint of pin to the center of the board (*R*_m_), as shown in Figure 7(a). Since these three magnetic pins without sufficient distance may rotate due to the magnetic repelling force from each other, the minimum distance between two pins (*X*_m_) without causing rotation needs to be identified first. Once *X*_m_ is determined, *R*_m_ can be calculated from the basic trigonometry. The transparent baseboard has three layers, as shown in Figure 7(b), where the top layer has three aligning lines and the lower two layers can hold the rotary joint of pin. There is a conductive ring on each layer to form the circuit for the LED indicator. Definitions of other dimensional parameters on the board are also illustrated in Figure 7(b).

**Figure 8:**
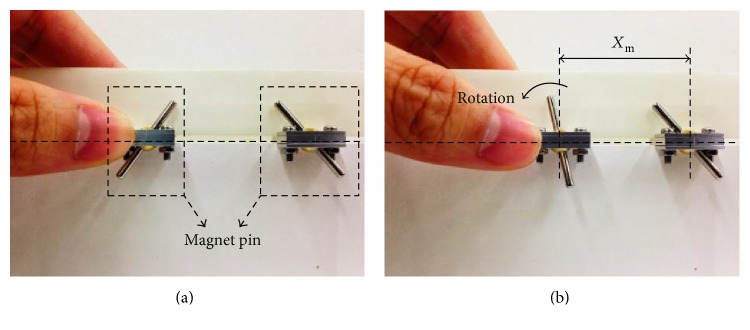
Experimental setup to determine *X*_m_: (a) initial state and (b) insufficient distance between two magnetic pins to cause interference.

The experimental setup to determine *X*_m_ includes two magnetic pins on a sliding track. The two magnetic pins made of NdFeB are identical with a length of 25 mm and a radius of 1 mm. First, one of the magnetic pins is fixed on the sliding track and the other pin is also placed on the track but away from the fixed pin, as shown in Figure 8(a). Then, the moving magnetic pin is pushed closer to the fixed one until the magnetic pins are affected by each other to have rotation, as shown in Figure 8(b). The measured maximum distance between two magnetic pins to have interference is found to be around 30 mm. Therefore, 31 mm is chosen as the lower limit of *X*_m_. According to the trigonometric function $\left(\sqrt{3}R_{m}=X_{m}\right)$, 17.90 mm is the lower limit of *R*_m_. Here, 18 mm is chosen to be the dimension of *R*_m_. Other designed dimensions of parameters are listed in Table 1.

Moreover, some orthopedic instruments may contain ferromagnetism material, such as r316 stainless steel. Usually the magnetic strength in orthopedic instruments is lower than the permanent magnet. It is suggested that the orthopedic instrument should be away from the device at least 30 mm during the targeting process.

## 4. Fabrication

**Figure 9:**
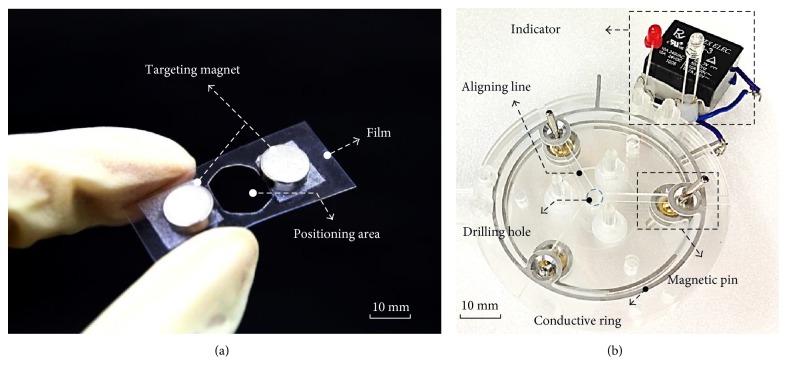
Photos of fabricated (a) 1st-stage targeting device and (b) 2nd-stage targeting device.

Figures 9(a) and 9(b) show the prototypes of the proposed 1st-stage and 2nd-stage targeting devices for this feasibility study, respectively. The 1st-stage targeting device consists of two targeting magnets on a flexible thin film with a hollow positioning area. The targeting magnets are made of NdFeB, and the flexible thin film is made of a 0.8 mm thick PET film. The 2nd-stage targeting device consists of three magnetic pins with rotary joints, a transparent baseboard with three conductive rings, and an indicator. The three layers to form the transparent baseboard are made of acrylic with a drilling hole and a conductive ring on each layer. On the top layer of the baseboard, there are three aligning lines, as shown in Figure 9(b). The three magnetic pins and rotary joints are made of NdFeB and Cu, respectively, and the rotary joint is assembled at the center of the pins.

## 5. Testing Results and Discussion

### 5.1. Accuracy Test of the 1st-Stage Targeting Device

**Figure 10:**
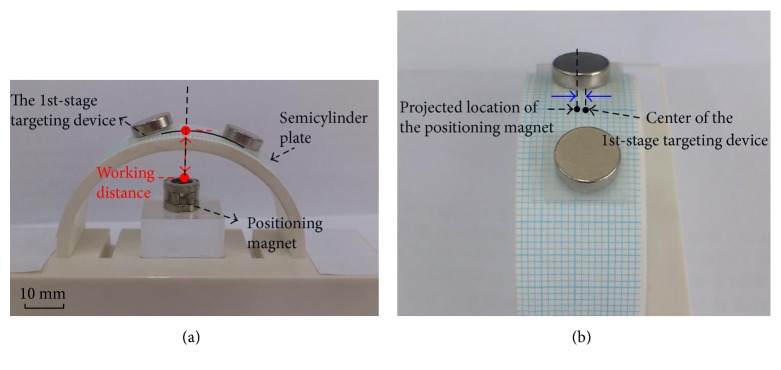
Measurement setup for the accuracy test of the 1st-stage targeting. (a) Side view and (b) top view.

**Figure 11:**
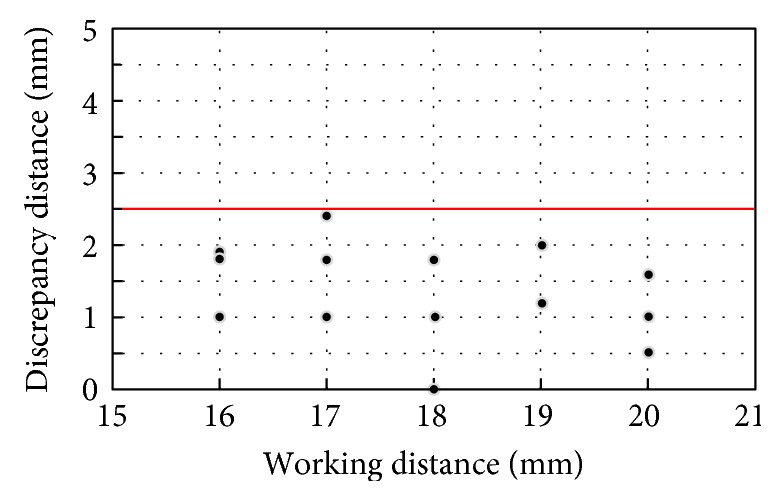
Accuracy test results on the 1st-stage targeting device.

The measurement setup to test the accuracy of the 1st-stage targeting device is shown in Figure 10, where the 1st-stage targeting device is placed on a semicylinder plate with a graph paper on it. The semicylinder plate having a 30 mm radius is used to simulate the curved shape of limbs. The positioning magnet is placed right beneath the middle of the curved plate. The test procedure involves the following three steps: (i) adjusting the working distance of the positioning magnet at a specified value, (ii) placing the 1st-stage targeting device on the semicylinder plate, and (iii) measuring the discrepancy distance between the center of the 1st-stage targeting device and the projected location of the positioning magnet on the graph paper, as shown in Figure 10(b). The working distance is tested at 16 mm, 17 mm, 18 mm, 19 mm, and 20 mm. At each working distance, the measurement is conducted three times, and the maximum discrepancy for different working distances is 1.9 mm, 2.5 mm, 1.8 mm, 2.0 mm, and 1.6 mm, respectively, as shown in Figure 11. The testing results show that the accuracy of the 1st-stage targeting is 2.5 mm, which confirms that a hollow positioning area with a 5 mm radius is sufficient.

### 5.2. Accuracy Test of the 2nd-Stage Targeting Device

**Figure 12:**
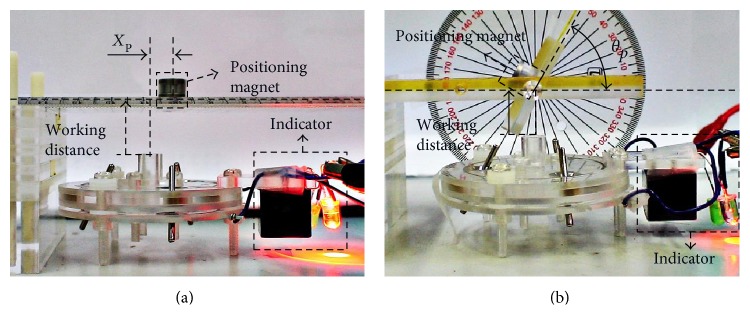
Measurement setup of the 2nd-stage targeting device for (a) translational and (b) angular accuracy tests.

There are two kinds of tests, translational and angular accuracy tests, to examine the guiding accuracy of the 2nd-stage targeting device on position and direction. The indicator with two LEDs is used to indicate the alignment (green light) or misalignment (red light) state directly. For the translational accuracy test, the positioning magnet is fixed on a ruler, as shown in Figure 12(a), where the 2nd-stage targeting device is placed opposite down and the ruler with the positioning magnet is above the 2nd-stage targeting device with different working distances. The translational accuracy test involves following three steps: (i) adjusting the working distance of the positioning magnet at a specified value, (ii) adjusting the horizontal distance (*X*_p_) between the positioning magnet and the projection location of the 2nd-stage targeting device, and (iii) recoding the state of the 2nd-targeting device by the indicator in each locations. For the angular accuracy test, the positioning magnet is fixed on a rotation table, as shown in Figure 12(b), where the 2nd-stage targeting device is also placed opposite down and the rotation table with the positioning magnet is above the 2nd-stage targeting device. The test process involves following three steps: (i) adjusting the working distance of the positioning magnet at a specified value, (ii) adjusting the angle of the position magnet by turning the rotation table, and (iii) recoding the aligned or misaligned state of the 2nd-stage targeting device by the indicator at each angle.

**Figure 13:**
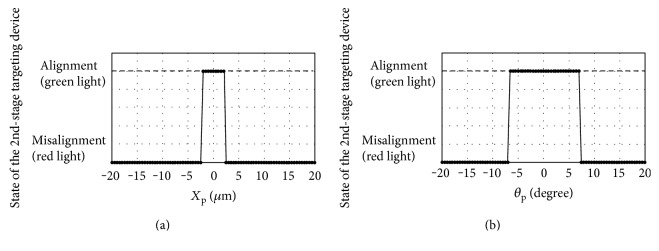
Experimental results of the 2nd-stage targeting device from the (a) translational and (b) angular accuracy tests.

The experimental results on translational and angular accuracy tests are shown in Figures 13(a) and 13(b), respectively. The working distance is also tested at 16 mm, 17 mm, 18 mm, 19 mm, and 20 mm. It is found that detection accuracy for alignment in position is ±2.1 mm and in direction is ±7 degrees.

### 5.3. Two-Stage Targeting Test and Positioning Time Measurement

**Figure 14:**
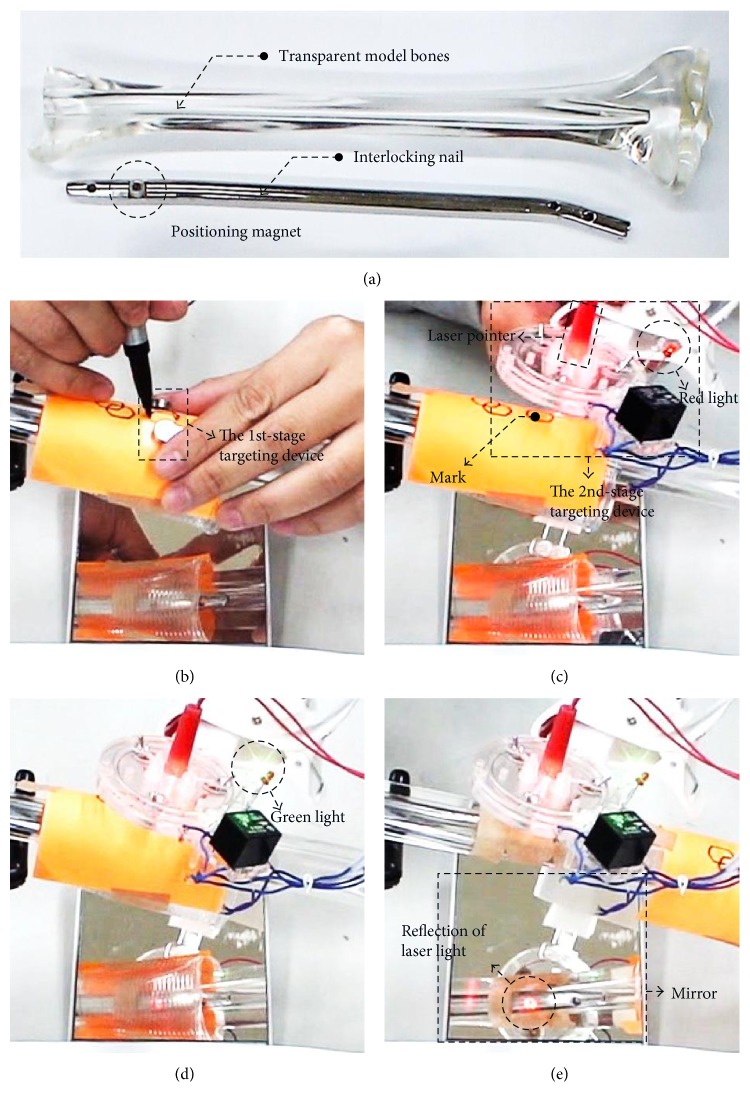
The two-stage targeting test on a transparent model bone. (a) A transparent model bone and a commercial interlocking nail with a positioning magnet fixed on the screw hole. (b) The 1st-stage targeting process. (c) The 2nd-stage targeting process in a misaligned state with red LED on. (d) The 2nd-stage targeting process in an aligned state with green LED on. (e) The paper barrier is moved away. Laser light successfully passes through the transparent model bone and the screw hole to the mirror to verify the success of the two-stage targeting process.

The two-stage targeting test combines the 1st-stage targeting process with the 2nd-stage targeting process to examine the successful rate and the time required for the whole targeting procedure. The positioning magnet is fixed on the screw hole of the nail to provide a magnetic field for targeting tests, as shown in Figure 14(a), and then, the nail with the positioning magnet is inserted into a transparent model bone. In the tests, the transparent model bone is covered by a paper barrier to ensure that the screw hole of the nail is invisible to the user. The 1st-stage targeting device then is placed on the barrier and moved around to find the location that the 1st-stage targeting device could be attracted on the paper barrier by the positioning magnet. After that, the positioning area is marked through the hollow circle on the 1st-stage target device, as shown in Figure 14(b). When the aiming direction is close to be horizontal, the 1st-stage targeting device may not stay on the surface due to the gravity. Therefore, the patient's pose needs to be adjusted to be more vertical in the 1st-stage targeting process. For the 2nd-stage targeting process, the 2nd-stage targeting device is moved around the positioning area identified by the 1st-stage targeting process. Before reaching the aligned state, the red LED is on, as shown in Figure 14(c). Once the device reached the aligned state, the green LED instead of the red one is on, as shown in Figure 14(d). In order to check the above targeting process is successful or not, a laser pointer is mounted on the drilling hole of the 2nd-stage targeting device. By moving the paper barrier away and turning on the laser pointer, when the alignment is truly achieved, the laser light should pass through the transparent model bone and the screw hole on the nail to the other side where a mirror is placed, as shown in Figure 14(e).

The two-stage targeting tests are conducted by two different persons with 25 attempts each, and 100% successful rate is achieved in all these 50 attempts. The time to finish the 1st-stage and the 2nd-stage targeting process is between 10–20 seconds and 29–215 seconds, respectively. The total time needed in the entire two-stage targeting process is between 43–230 seconds, less than 4 minutes.

Since the influence of gravity has been considered in the design of the 2nd-stage targeting device by using symmetrical magnetic pins with the rotary joints at the middle, the moment due to gravity on the magnetic pin can be balanced. Therefore, in the 2nd-stage targeting tests, even the aiming directions are randomly selected from horizontal to vertical directions, the successful rate still can reach 100% to verify that the gravity effect does not affect the accuracy at the 2nd-stage targeting process.

### 5.4. Drilling Test on a Swine Tibia

**Figure 15:**
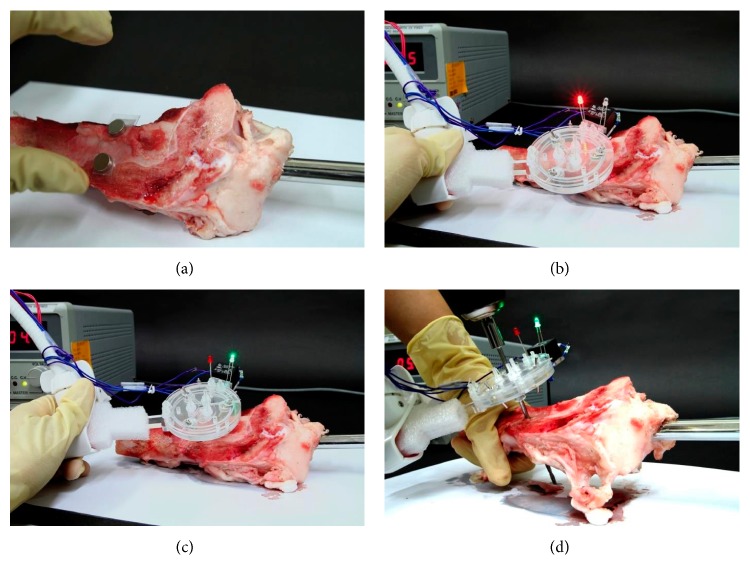
The drilling test with a swine tibia: (a) the 1st-stage targeting, (b) the 2nd-stage targeting process in misalignment state with red LED on, (c) the 2nd-stage targeting in alignment state with green LED on, and (d) successfully drilling through the screw hole of the interlocking nail inside the swine tibia.

In order to further verify that the proposed targeting devices can successfully identify the drilling position and direction, a drilling test on a swine tibia is also performed after the two-stage targeting process. The nail with the positioning magnet is inserted into the medullary cavity of the swine tibia, and the screw hole of the nail becomes invisible. The 1st-stage targeting is performed to find the positioning area for the 2nd-stage targeting process, as shown in Figure 15(a). In the 2nd-stage targeting process, before reaching the aligned state, the red LED is on, as shown in Figure 15(b). Once the aligned state is achieved, the green LED is on instead of red, as shown in Figure 15(c). Then, a powered drill with a 20 cm long drill bit is mounted on the drilling hole at the center of the transparent baseboard to successfully drill through the bone and the screw hole on the nail, as shown in Figure 15(d).

The two-stage targeting with drilling tests is conducted by two different persons with five attempts each, and 100% successful rate is achieved in all these 10 attempts. The time to finish the 1st-stage targeting process, the 2nd-stage targeting process, and the drilling process is between 7–11 seconds, 29–215 seconds, and 38–40 seconds, respectively, as listed in Table 2. The total time used in the entire process is between 95–300 seconds, less than 5 minutes.

## 6. Conclusions

Here, a novel two-stage magnetic targeting process with two passive targeting devices is proposed to provide a rapid, accurate, and secure method for the distal locking of interlocking nailing without radiation. The 1st-stage targeting device is used to focalize rapidly the screw hole area for the 2nd-stage targeting process. The 2nd-stage targeting device can accurately identify both the position and direction of the screw hole. From the accuracy tests, the experimental results show that alignment accuracy of the proposed 2nd-stage targeting device in position is ±2.1 mm and in direction is ±7 degrees. The results from the two-stage targeting tests show that the successful rate is 100% in 50 attempts. The time to finish the 1st-stage and the 2nd-stage targeting process is between 10–20 seconds and 29–215 seconds, respectively. The total time in the entire targeting process is less than 4 minutes. From the results of the drilling test on the swine tibia, 100% successful rate is achieved in all 10 attempts. The total time to finish the 1st-stage targeting process, the 2nd-stage targeting process, and the drilling process is less than 5 minutes. With the proposed targeting process and devices, the user could easily identify the alignment state in an intuitive way by direct observation through the transparent baseboard and the light-based indicator. The feasibility of the proposed two-stage passive targeting procedure to locate the screw hole position and orientation is successfully demonstrated. It is shown to be a time-saving, accurate, and radioactivity-free method in interlocking nailing with great potential on clinical purposes.

However, the proposed devices can be further improved, such as the working distance. Currently, the maximum working distance is around 20 mm, which may be sufficient for nailing surgery with thin soft tissue, such as tibia nailing, but not enough for nailing surgery with thick soft tissue, such as femoral nailing. In order to cover all cases in lower limb intramedullary nailing, the working distance of device needs to be further enhanced, such as using a stronger position magnet (a bigger position magnet or a magnet with a better magnetic material) or reducing the friction at the joints of magnetic pins to allow smaller magnetic force to rotate the pins.

## Figures and Tables

**Table 1:** Dimensions of the 2nd-stage targeting device.

Description	Parameter	Design size (mm)
The radius of the 2nd-stage targeting device	*R*	30
The thickness of each layer of the baseboard	*s*	2
The vertical distance between rotary joint and the baseboard	*h*	5
The radius of rotary joint of the magnetic pin	*R*_b_	3
The distance from the rotary joint of pin to the center of the board	*R*_m_	18
The diameter of the hole on the conductive ring	*d*	3.8

**Table 2:** Time used in the drilling tests.

	The 1st-stage targeting	The 2nd-stage targeting	Drilling	Total
Time (seconds)	7–11	91–251	38–40	95–300
